# Evaluation of a novel intervention to reduce burnout in doctors-in-training using self-care and digital wellbeing strategies: a mixed-methods pilot

**DOI:** 10.1186/s12909-020-02160-y

**Published:** 2020-09-09

**Authors:** Antonia Rich, Amira Aly, Marta E. Cecchinato, Laura Lascau, Magdalen Baker, Rowena Viney, Anna L. Cox

**Affiliations:** 1 Research Department of Medical Education, UCL Medical School, University College London, London, UK; 2 Computer and Information Sciences Department, Northumbria University, Newcastle Upon Tyne, UK; 3 UCL Interaction Centre, University College London, London, UK

**Keywords:** Doctors-in-training, Burnout, Wellbeing, Intervention, Boundary control, Digital technologies

## Abstract

**Background:**

Burnout for doctors-in-training is increasingly cause for concern. Our objectives were to assess the feasibility, acceptability and impact of a novel intervention to reduce burnout and improve wellbeing. This is the first wellbeing intervention for medical doctors to include strategies for work-life boundary management and digital wellbeing.

**Methods:**

Twenty-two doctors participated in face-to-face workshops which included group discussion of challenges experienced and strategies to enhance self-care and wellbeing. A pre-post-test mixed-methods evaluation was undertaken. Questionnaire measures were the Oldenburg Burnout Inventory, Warwick-Edinburgh Mental Wellbeing Scale and the boundary control subscale of the Work-Life Indicator (i.e., the degree of perception of control of the boundaries between work and personal life). Paired t-tests examined whether there were statistically significant differences. Eleven doctors also participated in post-intervention semi-structured interviews. Transcripts were analysed using thematic analysis.

**Results:**

The intervention was well-received, with all trainees finding the workshop useful and saying they would recommend it to others. At baseline most participants had scores indicative of burnout on both the disengagement (82%) and exhaustion (82%) subscales of the Oldenburg Burnout Inventory. One month post-intervention, participants had a statistically significant reduction in burnout (both disengagement and exhaustion) and improvement in boundary control. Wellbeing scores also improved, but differences were not statistically significant. Qualitative analysis indicated participants had welcomed a safe space to discuss stressors and many had implemented digital wellbeing strategies to manage their smartphone technology, and increased self-care such as mindfulness practice and walking in green space.

**Conclusions:**

The intervention reduced burnout and improved boundary control. We suggest that having protected time for doctors to share personal experiences, adopt digital wellbeing and self-care strategies are effective tools to support doctors’ wellbeing and should be investigated further.

## Background

### The impact of working pressures on doctors

Doctors in the NHS are under considerable working pressures which has a detrimental impact on their wellbeing. This is especially evident in trainees, many of whom do not have sufficient time to take breaks, eat and drink during their working day, and often work longer hours than rostered [1]. Over one fifth of doctors in training feel short of sleep while at work on a daily or weekly basis [2]. Doctors have high rates of mental health problems including depression, anxiety, drug/alcohol addiction, burnout and suicide [3]. Financial pressures within the NHS have resulted in staff being described as “shock absorbers”; working longer hours and more intensely to preserve patient safety [4] and heavy workloads seem unlikely to fall for the foreseeable future. Lack of work-life balance has increasingly been highlighted as a primary cause for current levels of stress in the medical profession, and for the increasing problems in recruitment and retention at all levels [5]. Digital technologies such as smartphones can also extend the time of availability to help and support colleagues beyond working hours, affecting one’s “digital wellbeing” [6]. We use the term “digital wellbeing” to refer to the emotional status that can derive from or be affected by the use of technology. There is a growing body of research in human-computer interaction and cyberpsychology looking at how the use of technology – and particularly its quest for our attention and availability – affects users. This can include healthcare professionals who make use of technology and particularly communication technologies to get and give support, find information and that acts as a vehicle for their availability outside working hours.

### Burnout and the “wellbeing paradox”

Despite being dedicated care-givers to others, doctors often do not engage in self-care behaviours themselves reflecting a “wellbeing paradox”, not getting sufficient rest and nutrition and failing to seek care for themselves when they are unwell [7–9]. The prevalence of burnout has been identified as higher in doctors than most other professional groups and the general population [10], however less than 1% of published literature on burnout focuses on doctors in training [11]. Burnout is characterised by emotional exhaustion, depersonalization and reduced personal accomplishment [12].

### Interventions to reduce burnout

A comprehensive systematic review and meta-analysis of interventions to prevent and reduce burnout identified that individual interventions such as mindfulness, stress management and small group discussions can reduce and prevent burnout [13]. The analysis covered 15 randomised trials and 37 cohort studies comprising a combined sample of 3630 doctors, found burnout decreased by 10% and concluded both individual and structural or organisational interventions can have clinically significant results [13]. The authors concluded “that these strategies can be effective approaches to reduce burnout domain scores” [13]. Thus, developing effective individual interventions is important to reduce and prevent burnout.

### Doctors’ mental wellbeing

Studies have generally focused on levels of stress, depression, addiction and burnout in doctors. This study measures burnout but also focuses on mental wellbeing and doctors’ positive mental health. There have been calls to measure physician wellness routinely, which has been described as a “missing quality indicator” [8]. There is limited research on doctors’ mental wellbeing and positive psychological functioning and understanding doctors’ psychological resources and whether these can be enhanced through interventions to provide a buffer from the stresses of training warrants attention. There is an increasing commentary that resilience and self-care need greater attention in medical education, beginning in medical school and continuing through post-graduate training [14, 15]. Unfortunately, while attention to this area is growing, medical education focusing on wellbeing currently tends to be the exception as opposed to the norm [14, 16, 17]. Redressing this has to be a priority.

### Work-life balance and microboundaries

Due to the demands of medical training, the boundaries between home and work can become blurred with trainees often sacrificing their limited free time for work and to study for exams, leading to reduced energy for coping with personal commitments [18]. Weak boundaries between work and non-work (such as family-related) tasks lead to repeated intrusions which are cognitively taxing and stress-inducing [19]. Further, modern technology creates fertile ground for repeated intrusions between work and non-work which is detrimental to work-life balance [20]. Boundary management [21] has the potential to reduce work-life imbalance, for example through microboundaries, which are digital “resilience strategies to help minimise transitions between different work and home roles and their associated negative effects” [6] and can improve one’s digital wellbeing [22]. Implementing microboundaries can free up cognitive and affective resources for recovery and potentially reduce work-life conflict [21]. An example of a microboundary is using a work phone and a personal phone, in this way separating the two domains [23]. Evaluation of microboundary strategies has found that they have been significantly effective in reducing stress and increasing boundary control [24].

### The intervention and aims of the study

Given doctors are time poor, we developed a brief intervention workshop for trainees to enhance their self-care skills and help them manage their work-life balance and use of technology. The workshop makes plain that doctors are resilient but that they are working in a system under pressure [4]. To develop the intervention, we draw on the research team’s experience of the challenges facing junior doctors [18], microboundary interventions [6, 23], and existing evidence regarding successful inventions to reduce burnout in doctors, such as mindfulness and small group discussions [13, 25].

The study has the following aims:

- Assess the feasibility and acceptability of an intervention to promote wellbeing among junior doctors;
- Measure the impact of the intervention on junior doctors’ mental wellbeing, burnout and boundary control, defined as the degree of perception of control of the boundaries between work and nonwork aspects of one’s life [20].

## Method

### Participants

The opportunity to participate in the workshops was advertised via email mailing lists, social media posts and posters placed in London hospitals. Fifty-six medical trainees signed up to participate and of these, 22 actually attended the workshops. To be included in the study, participants had to be in post-graduate medical training. Table 1 details participants’ demographics.

**Table 1:** Participant Demographics

Demographics	Number of Participants
Ethnicity	
White	9
Black and Minority Ethnicity	13
Gender	
Female	12
Male	10
Stage of Training	
Foundation	6
Core	4
Speciality Registrar	10
Prefer Not To Say	2
Primary Medical Qualification	
UK Graduate	20
International Medical Graduate	2
**Total Sample**	**22**

### Design

A mixed-methods longitudinal design was employed. Quantitative data was collected at two points: survey pre-workshop and 1 month post-workshop. Qualitative data was gathered during semi-structured telephone interviews (1–2 months post-workshop) using a phenomenological approach. The short-term follow-up time limits the threats to internal validity posed by history and maturation effects. Ethics approval was gained from University College London (reference: UCLIC/1314/003).

### The workshop intervention

The intervention was named iWARDS (**I**ndividualised **W**ellbeing **A**nd **R**esilience for **D**octor**S**). Workshops were facilitated by AR (Health Psychologist) and LL (Human-Computer Interaction Specialist).

The workshop consisted of two sections:

1. General wellbeing which included advice on self-care techniques (e.g., physical activity, eating habits, sleep strategies), self-compassion and mindfulness meditation.
2. Digital wellbeing which included advice on taking control of technology use through microboundaries to improve work-life balance. The microboundary strategies included: email management; notification and awareness cues management (e.g., disabling “read receipts” and “online status” in the social messaging app “WhatsApp”), and expectation of availability management (e.g., setting an out of office message for the weekends). More details about these strategies are available on the iWARDS website (https://iwards.wordpress.com/).

In addition to didactic instruction, the workshop included group discussion, experiential and reflective exercises. At the end of the workshops, participants were encouraged to set two SMART (Specific, Measurable, Achievable, Realistic, Timely) goals; one regarding self-care and the other regarding microboundaries. Refreshments were available and participants received a certificate of participation. Participants were given a copy of the iWARDS booklet containing the strategies covered in the workshops, which can be downloaded from the iWARDS website (available here: https://iwards.wordpress.com/).

### Measures

#### Burnout

The Oldenburg Burnout Inventory (OLBI) is a valid and reliable measure of burnout in healthcare workers [26]. OLBI measures burnout in two dimensions: disengagement (e.g., “I always find new and interesting aspects in my work”) and exhaustion (emotional, cognitive and physical exhaustion; e.g., “After working, I have enough energy for my leisure activities”). Each subscale consists of eight items, with four positively worded and four negatively. Responses ranged from 1 (strongly agree) to 4 (strongly disagree). Higher scores indicate greater burnout. Scores were averaged for analysis and a score of 2.25 or higher on exhaustion and 2.1 on disengagement are considered to be high, indicating burnout [27]. Cronbach’s alpha for the disengagement and exhaustion subscales were found to be 0.85 and 0.76 respectively.

#### Wellbeing

The Warwick–Edinburgh Mental WellBeing Scale (WEMWBS) is a validated and reliable 14-item measure of subjective wellbeing and psychological functioning [28]. Each item is measured on a 5-point Likert scale from 1 (none of the time) to 5 (all of the time) that best describes the previous 2 weeks, with a minimum score of 14 and a maximum of 70, with higher scores denoting better wellbeing. Examples include “I’ve been feeling interested in other people” and “I’ve been feeling good about myself”. Cronbach’s alpha showed good reliability (α = 0.89). The scale has been found to be sensitive for measuring change due to interventions with a change of three to eight points considered a meaningful improvement [29, 30]. The WEMWBS is included in the Health Survey for England, which found an average wellbeing score of 49.9 in 2016 [31].

#### Boundary control

Boundary control was measured through the boundary control sub-scale of the Work-Life Indicator (WLI) [32]. The sub-scale has three items, which have a 5-point Likert scale from 1 (Strongly Disagree) to 5 (Strongly Agree). Examples include “I control whether I am able to keep my work and personal life separate” and “I control whether I combine my work and personal life activities throughout the day”. Scores are averaged, and participants with ratings of 2 or less are classified as having “low boundary control” indicating that they have a sense of powerlessness over the interruption behaviours between their work and personal life. A score of three is considered “medium boundary control”. A score of four or higher is classified as “high boundary control” and denoted participants who have a sense of control of the interruption behaviours between their life roles [20]. The scale had good reliability (*α* = 0.91).

### Procedure

Trainees were recruited via medical education departments and social media (Twitter and Eventbrite). Upon sign-up, participants were sent an email link with the questionnaire containing the OLBI, WEMWBS, WLI measures and basic demographics, via the online survey platform Qualtrics. Six two-hour workshops were held in 2018 with 22 trainees in three London hospitals (University College London Hospital, The Whittington Hospital and The Royal Free Hospital). Following the workshop, trainees completed a feedback form to determine the usefulness and acceptability of the workshop and an online questionnaire 1 month post-workshop that included the OLBI, WEMWBS and WLI measures.

All participants were invited to take part in a semi-structured telephone interview 1 to 2 months later but not all responded. Most non-responders did not give a reason however one declined to participate due to heavy workload. Interviews focused on reflecting on the workshop in terms of what was most and least useful and their progress on goals set during the workshop. Eleven participants were interviewed and audio recordings were professionally transcribed verbatim. Interviews were scheduled to last approximately 30 min. The length of interviews ranged from 20 to 40 min with the average being 29 min. Data was analysed in NVivo 11.

Care was taken to ensure the quality of the qualitative research by employing the criteria of credibility, transferability, dependability, and confirmability as defined by Lincoln and Guba [33]. Three forms of triangulation were employed to establish credibility and confirmability. Firstly, data triangulation; data was collected at multiple sites and at different time periods. Secondly, method triangulation; multiple methods of data collection were employed, through the use of questionnaires and interviews. Thirdly, investigator triangulation; multiple researchers were used to code, analyse and interpret the qualitative data. Concerning reflexivity, an aspect of confirmability, the investigators discussed how personal and research values may influence the research. Care was taken for the researchers involved in qualitative analysis to have different professional backgrounds which included psychology (AR) and medicine (AA) thus bringing different perspectives to minimise potential bias. A further mechanism of confirmability and dependability was the engagement with an experienced qualitative researcher external to the study (RV, linguist) who examined the data and corroborated the interpretation, thus bringing an outside perspective to minimise potential bias. Regarding transferability, it is hoped the authors have presented in sufficient detail and transparently all aspects of the study for the reader to assess whether the findings would be transferable to similar contexts.

### Data analysis

#### Quantitative

##### Statistical analysis

Paired t-tests examined whether there was statistical significant change between pre-and post-intervention measures using IBM SPSS Statistics for Windows v.24 [34].

#### Qualitative

##### Thematic analysis of interview data

The interview transcripts were analysed using inductive thematic analysis [35]. Two researchers (AR and AA) read through all the transcripts and generated codes which were then used to produce an initial coding framework through discussion. A third researcher (RV) independent to the project team, with expertise in qualitative analysis was consulted to review the transcripts and framework to reduce the potential for bias. This framework was used by AR and AA, who coded three transcripts independently and discussed areas of disagreement and refined the framework where necessary. The framework was then used by AA to code the remaining transcripts. Both authors continued to meet throughout the iterative process to agree upon the themes and sub-themes which best captured trainees’ experiences.

## Results

### Quantitative results

Eighteen (82%) participants completed both pre- and post-surveys. At baseline, 18 (82%) participants had high disengagement scores (i.e. mean ≥ 2.1 on the OLBI-disengagement subscale) and 18 (82%) had high exhaustion scores (i.e. mean ≥ 2.25 on the OLBI-exhaustion subscale.) Ten participants (45%) had low boundary control, five (23%) had medium boundary control and seven (32%) had high boundary control. The mean wellbeing score of participants (M = 46.67, SD = 7.41) showed 13 (59%) had a wellbeing score lower than the mean reported by the Health Survey for England for the general population in 2016 (M = 49.9) [31].

#### Burnout

Mean level of exhaustion was significantly reduced from the pre-workshop (M = 2.78, SD = .43) to the post-workshop (M = 2.58, SD = .43); t(17) = 2.12, *p* = .049. Disengagement was also significantly reduced from participants’ pre-workshop scores (M = 2.43, SD = .47) to their post-workshop scores (M = 2.23, SD = .48); t(17) = 2.47, *p* = .02).

#### Wellbeing

Wellbeing scores increased from the pre-workshop (M = 46.67, SD = 7.41) to the post-workshop (M = 49.00, SD = 6.80), but this was not statistically significant t(17) = − 1.58, *p* = .13. However 41% (*n* = 9) of participants showed meaningful improvement in wellbeing scores (increase of between 3 and 8 points) [29, 30].

#### Boundary control

Boundary control scores significantly increased from the pre-workshop (M = 3.13; SD = .89) to the post-workshop (M = 3.63; SD = .84); t(17) = − 2.21, *p* = .04).

#### Participant feedback

The intervention was well-received with all trainees finding the workshop useful and saying they would recommend the workshops to others. 67% of the participants responded that they were “very likely” to implement any of the strategies they heard in the workshop, and 33% of the participants reported that they were “somewhat likely”.

### Qualitative results

Thematic analysis revealed the following seven themes:

1. Opportunity for reflection and to prioritise wellbeing
2. The value of sharing and hearing the experiences of others
3. Workshop content
4. Self-care goals and outcomes; enablers and barriers to implementation
5. Microboundaries goals and outcomes; enablers and barriers to implementation
6. Future intentions
7. Improvements for future workshops

Illustrative quotations for each of the Themes can be seen in Table 2.

**Table 2:** Illustrative quotations from interviews

Theme	Illustrative Quotation
**1: Opportunity for reflection and to prioritise wellbeing**	*“I think there is still, sometimes, difficulty communicating with your colleagues. I think there is still a sense of machoism about it” (P19)*
	“W*hat I found useful is that someone, anyone, is just acknowledging the stresses that are there” (P5)*
	*“I think about how you need to stop and think about yourself as well and don’t think about the patients only” (P1)*
	“*Normally you just carry on because there’s no time to think about it” (P2)*
	*“When I’m not at work I spend a lot of time absolutely exhausted so I don’t stop and think about things. It’s not that I couldn’t, I just don’t have the energy for it. I just sit and watch TV or whatever” (P17)*
	*“I think it makes you reflect on yourself. It made me realise better that the weeks when I feel really crap are the weeks when I don’t put any care attention at all on my balance. So that’s probably the main thing I learnt about myself” (P17).*
	*“It, sort of, prompted me to think about myself and certain things and what I wanted to change and what may be contributing to some of my stresses” (P2).*
**2: Sharing and hearing experiences of others**	*“I think it was like a safety room where you can actually express yourself and no one’s going to judge you or take whatever you’re saying and take it somewhere else” (P1)*
	*“You know, there’s an expectation that we are supposed to be very resilient and just be able to cope with whatever…. I can probably identify five doctors who’ve committed suicide. And actually, you know, I feel nobody should have to allow themselves to get to that point in life without being able to talk and look for help. And so I think it was really important… that we had that opportunity to talk about our own personal experiences with others. Because what you quickly realise is that actually everyone’s saying we could all relate to, we’ve all experienced it, even when we’ve had to put on a tough face at work and pretend the abnormal is normal” (P20).*
**3: Workshop content:** **Self-care**	*“The most useful stuff I found, about being aware of what the stresses can be and how to manage it”* (*P2)*
	*“I actually really liked the meditation techniques. I thought they were really helpful” (P9).*
	*“I was actually doing quite a bit of work myself. You know, like self-help material reading. So, I didn’t find that aspect as helpful. But … I think the definitions were helpful. Kind of understanding what it actually means to be compassionate towards yourself, and what does wellbeing mean.”* (*P21).*
**3: Workshop content: Microboundaries**	*“So, when I am at home it’s quite stressful because I’ll get an email through about work stuff… So, yes I think the concept of having separate emails. And what I’ve done differently as a result is, I don’t necessarily open the ones that don’t look that kind of acute, I suppose”* (*P4).*
	*“Just showing us how to turn off notifications or set them for, you know, not to be on between 9 and 5 or whatever, that was the most useful bit I think”* (*P5).*
**4: Self-care goals and outcomes; enablers and barriers to implementation**	“*I’m calmer and I feel like I’m bringing back that equilibrium that I had before…and being cheerful and improving how you feel about yourself” (P1).*
	*“I really did take away that job, and setting own goals, and making it a SMART goal. And making us actively aim for something, and put it into action. That was quite helpful, because I felt motivated to see that through” (P21).*
	*“I think it is [mindfulness] very relaxing and I think it has been making it easier for me to get to sleep. The times I have found it difficult is when I have been doing night shift, obviously, because you get out of your routine” (P12).*
	*“So, the self-care one was to join up with an exercise class. So, I had every intention, I did my research, found the exercise class I wanted to go to. It’s on Thursday evenings. My timetable changes, I now can’t go” (P5).*
	*“So I said I want to be more active, and I want to go to the gym more often…but it’s that I haven’t been able to do it over some months before that because I’d had exams,* etc.*” (P9).*
**5: Microboundary goals and outcomes; enablers and barriers to implementation**	*“It sort of made me put myself in control of my time and space” (P20); “My time at work is more productive…I’m much more efficient in my administrative time now” (P5)’.*
	*“For me if I tried to make too many changes I’d just forget about all this. Whereas if I committed to one and focused on that fully…then I have a higher likelihood of achieving that rather than try to do too many at once” (P2).*
	*“Because it’s quick, isn’t it, it doesn’t require a big-time commitment”* (*P5).*
	“*I feel like I should be available* [out of hours] *if there’s something important” (P12).*
	*“My consultant 1 day had a huge upset with me, because I wasn’t responding to my mobile phone, she was texting or calling me or whatever at work but I was in teaching. But if I turned off the notification, you know, I can’t really go around to my consultant and say, hey, just… I’m letting you know this is what I’m doing. She’ll be saying, well no, you should keep them on and you should ignore all the personal messages. But that’s incredibly hard to do” (P5).*
**6. Future intentions**	*“I mean, if I’ve done it for the last few weeks now, it’s easy now I’ve got into to the habit” (P1).*
	*“I think I’m going to just talk to a few more people. And maybe even just get some psychotherapy for it, to be honest. So just generally, because it’s… I think it’s still an on-going thing. You know doctors always tend to be very self-critical” (P21)*
	*“I think it’s more about the general concept that I’d like to continue. I’d like to think that when I’m a consultant that I’d like to value a lot of these things and I’ll make it known to my juniors I hope. And my team that it’s really needed”* (P4).
**7. Improvements for future workshops**	“*It would’ve been useful to have an extra ten, 15 min so that anyone who wants to do that [implement the changes immediately] maybe stays at the end and gets some support, like, I suppose tech support to try and just do that” (P5).*
	*“I think the online thing is even better because then no one has to commute somewhere. They can do it at their own time, over the weekend or maybe midnight” (P1)*

### Theme 1: opportunity for reflection and to prioritise wellbeing

Trainees referred to the challenges of discussing problems in their normal working environment, where medical culture is one where there is little open discussion about challenges to wellbeing*.* Doctors are expected to be “*hardened*” *(P1)*, *“resilient” (P20)* and not *“admit any weaknesses” (P4)*. Thus, stresses are not openly vocalised, and the workshops provided a welcome respite. For several trainees a useful aspect was an acknowledgement that wellbeing was a challenge for doctors, and simply hearing the message that they should take care of themselves was valuable.

The workshops provided time and space for self-reflection, which was typically not possible due to the intensity of work and/or due to simply being exhausted, there was not the mental energy available. Through dedicated time, the workshops gave doctors the opportunity to develop greater self-awareness of the impact of work on their wellbeing, the importance of self-care and motivation for change.

### Theme 2: sharing and hearing experiences of others

The structure of the workshop involved interactive exercises, sharing experiences and listening to one another. The workshops created a safe space where trainees could speak openly about their experiences, without fear of being judged. The expectation that doctors should be resilient was discussed by one doctor in light of colleagues who had sadly committed suicide, highlighting the importance of a safe place where personal experiences could be discussed openly.

### Theme 3: workshop content

#### Self-care

The positive aspects from the self-care content of the workshop were that participants reported increased awareness of the value of self-care, enhanced self-awareness and tools to manage stress. Examples of strategies noted by participants as being useful were mindfulness techniques, creating a “no-list”, and physical activity.

In terms of the least useful aspects, two participants were already familiar with the material presented.

#### Microboundaries

Most participants commented on the usefulness of creating stronger boundaries between work and non-work roles with the use of technology. Doctors commented on the practical tips they had gained from the session. This tended to be with their smartphone and/or email.

### Theme 4: self-care goals and outcomes; enablers and barriers to implementation

#### Goals and outcomes

Ten out of the 11 participants who were interviewed made self-care commitments. Many of the self-care behaviours participants had adopted were regarding physical activity (e.g., walking in green spaces). Two participants chose to adopt mindfulness. One participant made a commitment to be more self-compassionate*,* and another participant had developed a "no-list" and set greater boundaries at work*.* Participants described positive outcomes as a result of changes in their self-care behaviours. The benefits were wide-ranging, including feeling more relaxed, happier and experiencing an increase in self-esteem from a sense of achievement.

#### Enablers to implementation

Several factors facilitated the implementation of commitments: setting SMART goals, writing down a commitment during the session, and scheduling an activity in their diary.

#### Barriers to implementation

Not all participants were able to keep their commitments. The nature of having to adhere to a rota, changing work patterns and a heavy workload presented challenges to the implementation of commitments for a few participants. One participant started practicing mindfulness prior to going to bed which had been beneficial, but maintaining this routine during night shifts has been challenging*.* Another participant’s intentions were not met because of a change in their rota, meaning that they could no longer attend their exercise class as planned. For another participant, the demands of exams had interfered with their physical activity goal.

### Theme 5. Microboundary goals and outcomes; enablers and barriers to implementation

#### Goals and outcomes

The most popular microboundary goals concerned social media and messaging apps (e.g., WhatsApp). Goals included disabling notifications to reduce interruptions, turning off awareness cues (e.g. read receipts) to manage expectations of availability, and device management to reduce access to or appeal of social media apps (e.g. moving all social media apps in a folder away from the home screen). Participants reported a myriad of positive outcomes as a result of implementing microboundary strategies including reduced stress, better boundaries between work and personal life, increased productivity and increased control.

#### Enablers to implementation

Several factors contributed to participants’ success in implementing microboundaries: setting the intention and getting started, choosing one achievable goal and due to the fact that microboundary strategies are quick to implement.

#### Barriers to implementation

Some doctors felt that they needed to be constantly available on their devices as they were worried they might miss out on important work or personal matters. Another doctor, expressed concerns, “*in anticipation*” *(P5)* about interpersonal difficulties arising from expectations of availability not being met as this had been their experience before even when they have been unable to respond because of other responsibilities.

### Theme 6. Future intentions

Many of the participants expressed the desire to continue with the strategies they found useful, where the changes had already become routine. For some participants, the workshops stimulated deeper reflection, prompting them to consider seeking psychotherapy. Another participant discussed the value of self-care and wanting to embed it into their work for the future when they became a Consultant, expressing the desire to communicate the value of self-care to their junior doctors.

Overall, 94% (17/18) of participants were interested in attending further work-life balance training and 89% (16/18) expressed interest in further mindfulness and self-care training.

### Theme 7. Improvements for future workshops

A couple of participants expressed a desire for more practical instruction regarding how to implement the microboundary strategies, suggesting they would have liked to have implemented some of the microboundary strategies on their electronic devices during the workshop but had been unable, mentioning lack of sufficient time and detailed instruction as obstacles. Participants were asked whether they would like the intervention to be available online, such as via an app and were generally keen for this alternative, given the difficulties of attending face-to-face workshops due to lack of time.

## Discussion

This is the first known study that has integrated education about the use of microboundaries for digital wellbeing [22] with more traditional stress-reduction techniques such as mindfulness and self-care strategies, self-compassion and physical activity, in a wellbeing intervention for doctors in training. Prior to the intervention the majority of trainees reported symptoms of burnout. Post intervention, there was a statistically significant improvement in boundary control and in both the emotional exhaustion and disengagement components of burnout, although not for mental wellbeing. Previous work which evaluated microboundary strategies, found a significant reduction in perceived stress, along with a significant increase in boundary control [24]. One possible explanation for the non-significant result in this study is that mental wellbeing encompasses several aspects, not just the absence of stress, as defined by WHO [36]. Triangulation with the qualitative findings provides insight into the factors contributing to the success of the intervention. Trainees described their working environment as a culture where challenges to wellbeing are rarely discussed, with an intense workload that resulted in little time and energy for reflection on their stressors. The workshops were welcomed because they provided a unique opportunity to hear the explicit message that doctors need to take care of themselves and by providing time and space which gave the opportunity to reflect on their wellbeing, stressors and identify areas they would like to change. Being in a non-judgemental environment, and participating in group exercises which involved sharing and hearing experiences of others, was highly valued. Given their working environment tends not to welcome open communication about perceived weaknesses, sharing experiences openly with other trainees in a non-judgemental, safe space was felt to be particularly important.

Microboundaries were the most frequently implemented strategies, particularly in regards to managing smartphones (e.g. disabling notifications), and the most popular self-care strategies implemented were physical activity (e.g. walking in green spaces) and mindfulness. Interviews revealed that strategies such as turning off notifications from social media resulted in fewer interruptions, with both affective (e.g. reduced stressed) and functional benefits (e.g. better time management). This is in line with previous empirical work which has shown that removing email notifications leads to stress reduction [37]. Positive outcomes from increased self-care behaviours included feeling more relaxed, happier and an increase in self-esteem.

Enablers to the successful implementation of commitments included writing down their commitment during the workshop and planning the activity, such as scheduling time in their diary and setting SMART goals. Goal setting is known to be an effective behaviour change technique [38]. Goals which tended to be successful were those that were quick and easy to implement, perhaps unsurprisingly as doctors are time poor. Due to experiencing the benefits of these changes, many expressed a desire to continue to implement the changes in the future. Not all commitments were successful however. Concerning microboundaries, some felt they should always be available, either because of their responsibilities or due to expectations of others, such as their consultant. This is in contrast to previous work, which suggests that once smartphone settings are changed (e.g. removing notifications), these changes are kept long-term, even 2 years later [39]. Findings from the study presented in this paper suggest that for particular professions that rely on on-call availability and with caring responsibilities, it is crucial for users to be fully in control of how microboundaries are implemented and adapted over time to meet their needs. With self-care, some commitments, for example, attending an exercise class, had not been possible because of rotas and changing work patterns. In terms of future interventions, one improvement would be to offer practical assistance for participants who wished to implement microboundary strategies immediately after the workshop. Another would be to explore the possibility of an online intervention via an app or website, which would allow doctors to access the intervention at a time convenient to them.

Whilst the results of the intervention are encouraging, burnout levels for both emotional exhaustion and disengagement remained high as classified by the Oldenburg Burnout Inventory suggesting that significant levels of burnout existed in participants post-intervention. It should be remembered that interventions targeting the individual to increase wellbeing and reduce burnout cannot be the only solution. Such interventions focusing on the doctor alone, run the risk of placing responsibility for good mental health on the doctor themselves, while neglecting the organisational and structural context in which they are operating [25, 40]. That organisational and structural factors require change is beyond doubt, but the unfortunate reality is that doctors currently practicing in the NHS will be working in a system that is under pressure [4] for the foreseeable future and organisational level change typically happens at a glacial pace, especially if it is to be sustainable [41]. Both interventions that target the individual (e.g. mindfulness) and organisational factors (e.g. work environment) are required; both of which produce similarly large improvements in burnout [13].

### Strengths of the study

This is the first study that has used microboundary techniques to increase wellbeing and reduce burnout in trainee doctors integrated with other techniques with prior evidence of effectiveness including mindfulness and group discussion [13]. In addition to the originality of the intervention design, strengths include the high response rate for completing the follow-up questionnaire (82%). Doctors' response rates to questionnaires can be low (e.g., [42]) and inability to follow up problematic due to potential selection bias and loss in sample size. The questionnaires chosen were brief but also sensitive to change in a small sample, with the exception of the WEMWBS.

The use of both quantitative and qualitative methods provides a more comprehensive understanding of participants’ experiences and intervention effectiveness, in addition to enhancing the rigour of the study [43, 44].

### Limitations of the study

Limitations include lack of a control group, meaning we cannot exclude other possible confounding factors which could have influenced post-workshops scores. Multiple channels were used to maximise recruitment but despite trainees signing up, there were challenges with attendance, with only 51% of those signing up actually attending the sessions. Cancellations were frequently to do with work pressures, such as staff shortages and needing to work late. This suggests wellbeing interventions should not be an “add-on” to an existing schedule but part of routine medical education. The small sample size limits the generalisability of the findings and could have resulted in the lack of a detected effect for improvement in mental wellbeing scores, which were not statistically significant (type II error). Participants were a self-selecting sample, and may have been attracted to the workshop because of higher rates of burnout. However the GMC’s National Training Survey which includes data from over 50,000 UK doctors, suggests our sample was not unique, with 39% reporting that their work is emotionally exhausting and 56% reporting that they were always or often worn out at the end of the working day [45]. Follow-up data was collected 1 month post intervention so it is unknown as to whether the improvements in burnout and wellbeing would be sustainable over a longer period of time. Future research could address these limitations with a larger sample, an experimental design and a follow-up period of a longer duration.

## Conclusion

Despite high rates of burnout, interventions to enhance wellbeing and reduce burnout are not typically included in medical education. This pilot intervention suggests trainee doctors would benefit from self-care and digital wellbeing strategies and further research could explore the potential for this intervention in a larger sample of doctors with a view to becoming a routine part of training.

## Data Availability

In order to protect the confidentiality of research participants, the data generated and analysed during the current study are not publicly available.

## Abbreviations

iWARDS: Individualised Wellbeing And Resilience for DoctorS
OLBI: Oldenburg Burnout Inventory
NHS: National Health Service
SMART: Specific Measurable Achievable Realistic Timebound
WEMWBS: Warwick–Edinburgh Mental WellBeing Scale
WHO: World Health Organisation
WLI: Work-Life Indicator
